# False Positive Dobutamine Stress Echocardiography Induced by Esmolol

**Published:** 2014-01-01

**Authors:** Ali Hosseinsabet, Arshia Shahmohamadi Mosavi

**Affiliations:** 1 Cardiology Department, Tehran Heart Center, Tehran University of Medical Sciences, Tehran, IR Iran; 2 Cardiology Department, Shaheed Hasheminejad Kidney Center, Iran University of Medical Sciences, Tehran, IR Iran

**Keywords:** Stress Echocardiography, Dobutamine, Beta-Antagonists

**Dear Editor,**

Dobutamine stress echocardiography is one of the several noninvasive methods for detection of coronary artery disease (1). New regional wall motion abnormalities have been reported in dobutamine stress echocardiograpghy after intravenous beta blocker administration (2, 3) Thus, this method has been recommended for increasing the test sensitivity (4). The sensitivity, specificity, accuracy, and positive and negative predictive values of dobutamin stress echocardiography without beta blocker have been reported to be 84%, 92%, 87%, 95%, and 77%, respectively. Using beta blocker, on the other hand, these measures have been reported as 92%, 89%, 91%, 94%, and 87%, respectively (3). In theory, beta blocker induced false positive dobutamine stress echocardiography can occur (5).

In this study, a 49 year old woman with the history of abnormal uterine bleeding was candidate for abdominal hysterectomy. The patient complained about atypical chest pain without any defined coronary artery disease risk factors, and was referred for dobutamine stress echocardiography evaluation for preoperative risk stratification. In physical examinations, the patient’s heart and lung had unremarkable findings and other cardiovascular examinations were normal. The vital signs before dobutamine stress echocardiography were heart rate = 88 bpm, blood pressure = 135 / 80 mmHg, and respiratory rate = 15. Besides, electrocardiography showed normal sinus rhythm with no ST-T changes. Additionally, resting echocardiography showed normal Left Ventricular Ejection Fraction (LVEF) (LVEF = 55%) with no regional wall motion abnormalities, mild LV diastolic dysfunction, and no significant valvular diseases. Dobutamine doses were increased step by step to 40 mic / Kg / min and the heart rate increased to 145 bpm (90% maximal predicted heart rate), blood pressure increased to 140 / 80 mmHg with no regional wall motion abnormalities, and LVEF increased to 65%. Then, Esmolol 0.5 mg / Kg was administrated in one minute (6). Thereafter, the heart rate decreased to 77 bpm, blood pressure decreased to 130 / 80 mmHg, inferior and inferoseptal walls appeared to be hypokinetic, and LVEF decreased to 45 - 50%. It should be mentioned that the patient was asymptomatic during the procedure without any ST segment changes.

**Figure 1.**
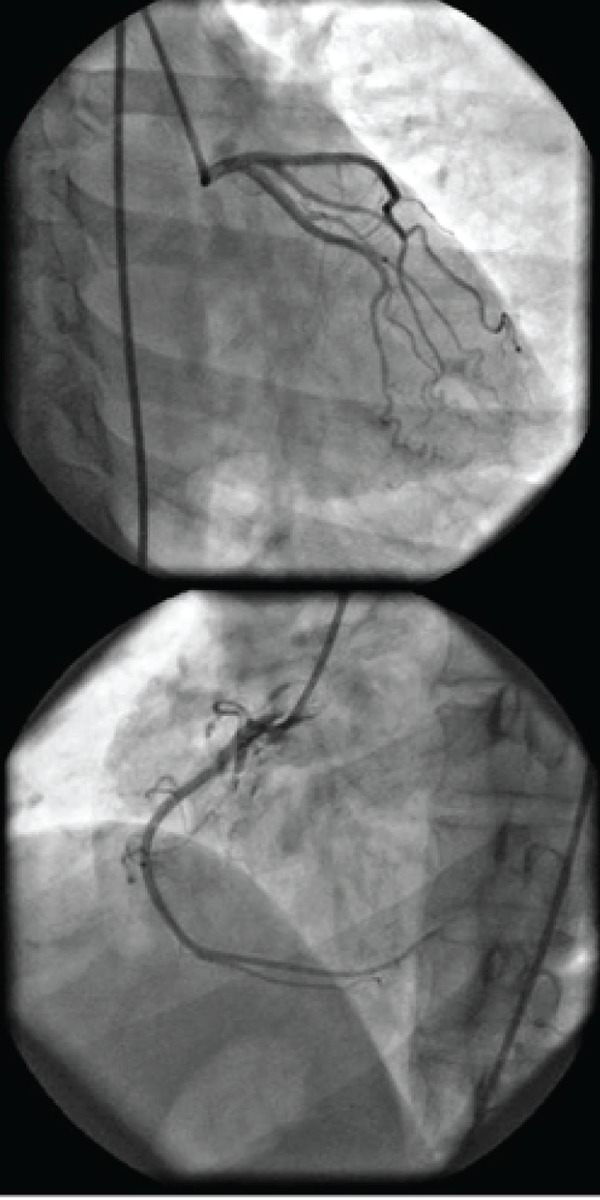
Left and Right Coronary Arteries Seem to Be Normal

**Figure 2.**
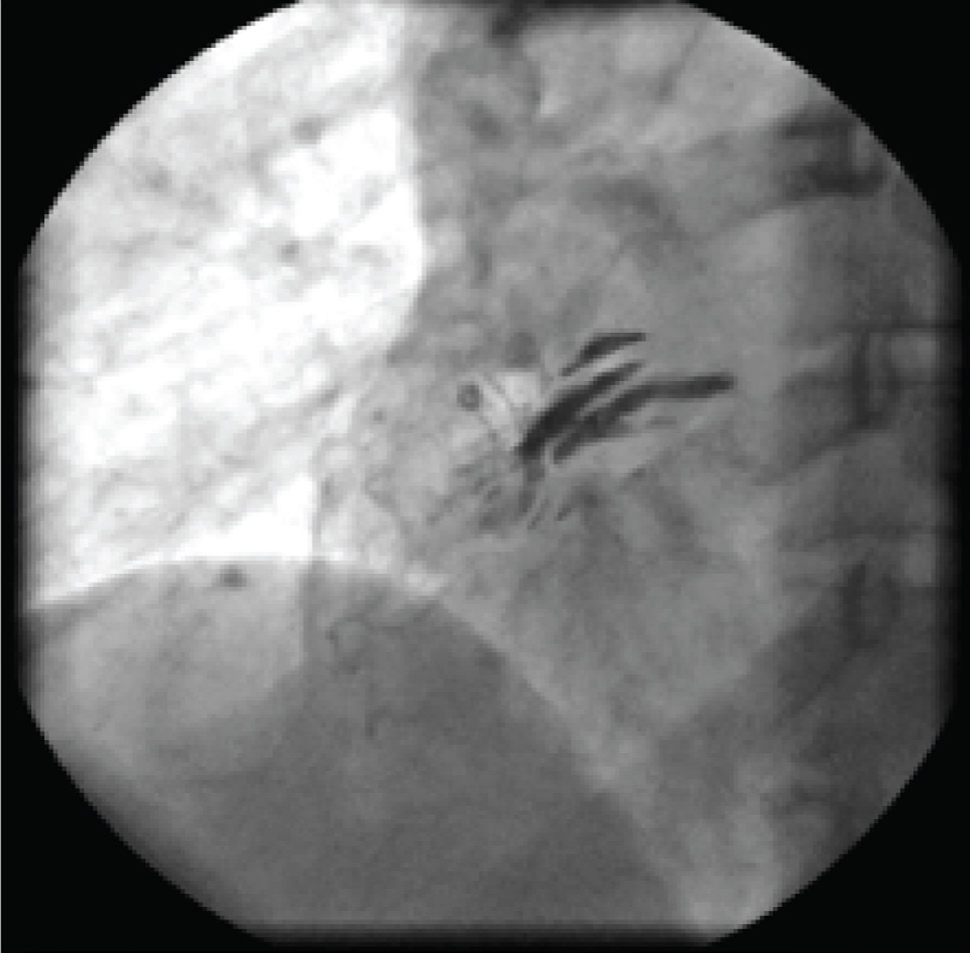
Dye Stasis in the Ascending Aorta Wall Suggestive of Dissection of Aorta

According to dobutamine stress echocardiography results, the patient was referred for selective coronary angiography. In selective coronary angiography, the left coronary system was normal. The right coronary artery was also normal; however, when the catheter was engaged in the right coronary artery and injection was done, localized dissection of ascending aorta appeared (Figures 1 and 2). The patient was totally asymptomatic without any chest pain and with stable hemodynamic. Thus, she was admitted to the coronary artery unit (CCU) for more observations and evaluations. Two days after catheterization, CT angiography of the aorta was carried out revealing very small localized dissection of the aorta. The patient was totally asymptomatic with hemodynamic stability in the course of admission. Serial electrocardiograms and transthoracic echocardiographies also indicated no new changes since early echocardiography examination. Finally, the patient was discharged at the fifth day of admission. She also had no new complaints and was unremarkable in physical examinations during the follow-up visits.

Dobutamine stress echocardiography is a useful method for detection of coronary artery disease and beta blocker administration is believed to increase the sensitivity of this method. This might be due to better visualization of new wall motion abnormalities that occur at peak stress (2, 3). Another proposed mechanism is that dobutamine stimulates beta 1, beta 2, and alpha 1. By administration of beta blockers, beta receptors are inhibited and unopposed alpha receptors can induce coronary vasoconstriction and decrease coronary flow reserve; therefore, wall motion abnormality can be induced (2, 3). An extreme side of the second mechanism can be the occurrence of coronary vasospasm induced by acute beta receptor blockage by esmolol during high doses of dobutamine as well as alpha receptor liberation. This could also explain our findings. In fact, increased coronary artery vasoconstriction can lead to an increase in coronary resistance and a decrease in coronary artery blood flow, myocardial ischemia, and new regional wall motion abnormalities. Mathias W Jr et al. (3) briefly reported one false positive dobutamine stress echocardiography resulting from beta blocker usage. Their case had lateral wall motion abnormality in the recovery phase after metoprolol administration and presented with 40% stenosis in diagonal artery in coronary angiography. However, our case had more extensive regional wall motion abnormalities and normal coronary artery.

Beta blocker usage in dobutamine stress echocardiography is recommended for increasing the test sensitivity, while false positive results can be encountered due to coronary vasospasm. Hence, this possibility should be taken into account while interpretation of dobutamine stress echocardiography.
